# Fruit and vegetable intake and vitamins C and E are associated with a reduced prevalence of cataract in a Spanish Mediterranean population

**DOI:** 10.1186/1471-2415-13-52

**Published:** 2013-10-09

**Authors:** Maria Pastor-Valero

**Affiliations:** 1 Departamento de Salud Pública Historia de la Ciencia y Ginecología, Universidad Miguel Hernández, Elche, Spain; 2 CIBER en Epidemiología y Salud Pública (CIBERESP), Madrid, Spain

**Keywords:** Cataract, Fruit intake, Vegetables intake, Antioxidant vitamins, WHO recommendations

## Abstract

**Background:**

Cataract is among the major causes of vision impairment and blindness worldwide. Epidemiological studies support the role of antioxidants in the etiology of cataract, but the evidence for one specific antioxidant over another is inconsistent. Few studies have examined the association of cataract with fruit and vegetable intake with inconclusive results. In the present study, the relationship between cataract and fruit and vegetable intake and dietary and blood levels of carotenoids, vitamins C and E were examined in a Spanish Mediterranean population.

**Methods:**

The present work is an analysis of data from 599 elderly ( ≥ 65 years) participants from the Spanish segment of the EUREYE study. This is a European multi-center cross-sectional population-based study. Cataract was diagnosed using a slit-lamp examination and defined as any lens opacity in either eye or evidence of its removal (cataract extraction). Energy-adjusted intake of fruit and vegetables and antioxidant vitamins was estimated using a semi-quantitative food frequency questionnaire. Plasma concentrations of vitamin C were analyzed by a colorimetric method and carotenoids and α-tocopherol by a HPLC method. The associations between cataract and quartiles of fruit and vegetable intake and plasma antioxidants were investigated using logistic regression models.

**Results:**

Of the 599 elderly recruited, 433 (73%) had cataract or cataract extraction, 54% were women and 46% were men. After adjustments, increasing quartiles of combined fruit and vegetable intake were associated with decreasing reduction of odds of cataract or cataract extraction, (*P for trend* = 0.008). Increasing quartiles of dietary intakes from 107 mg/d of vitamin C showed a significant decreasing association with prevalence of cataract or cataract extraction (*P* for trend = 0.047). For vitamin E, a protective association was found from intakes from 8 mg/d, but no linear trend was observed across quartiles of intake (*P* for trend = 0.944).

**Conclusions:**

High daily intakes of fruit and vegetables and vitamins C and E were associated with a significantly decreased of the prevalence of cataract or cataract surgery. This study reinforces the WHO recommendations on the benefits of diets rich in fruit and vegetables.

## Background

Worldwide age-related cataract (ARC) is the leading cause of low vision and blindness, accounting for almost half of all cases [1]. There is no data on the prevalence of cataract in Spain, but estimates based on a systematic review of US, Australian and European population-based studies describes increasing prevalence with advance age ranging from 15% to 30% in individuals by the age of 50 and 40% to 60% among those over the ages of 70 or 75 [2].

Opacities of the lens occur primarily due to age as a direct result of oxidative stress. In this framework of ageing, ARC is considered to be the result of lifelong molecular damage by reactive oxygen species to lens proteins [3]. To counteract these potentially damaging stress factors, the lens has an elaborate antioxidant defence system including enzymes such as glutathione peroxidase, glutathione reductase, catalase and superoxide dismutase, and the antioxidant vitamins C, E and some carotenoids [4-6]. These antioxidants cannot be synthesized by the body and must be obtained from dietary sources, mainly fruit and vegetables. Because of their concentrations and antioxidant activities in the lens, most epidemiological studies have focused more on the role of vitamins than on that of foods [7]. The results from these observational studies have supported a role for individual antioxidants. However, the evidence for one specific antioxidant over another is inconsistent. In fact, the lack of clear evidence from high quality RCTs of antioxidant supplementation on cataract, has led to claims that the evidence regarding antioxidants from observational studies is confounded by other factors associated with healthy diets [8,9]. A number of studies have examined the association of cataract with foods [10-16] or with quality or type of diet [16-18].

In 2004, the WHO launched a strategy to reduce the prevalence of non-communicable diseases [6]. One of the dietary recommendations is to eat 5 or more portions of fruit and/or vegetables a day ( ≥ 400 g/day). Few studies have examined fruit and vegetable intake in relation to the risk of developing age-related cataract. The results have been inconclusive [11,16], and only one used prospective data [9].

In order to determine the relationship between fruit and vegetable intake and concentrations of antioxidant vitamins with cataract prevalence and/or cataract surgery in an elderly Mediterranean population, Spanish data from the EUREYE study were analyzed.

## Methods

### Study population and sample

This study analyzed data from the Spanish segment of the European Eye study (EUREYE) collected between February 2000 and November 2001. EUREYE is a multicenter population-based cross-sectional study aimed at estimating the prevalence of AMD (age-related macular degeneration) and associated factors, including diet, in an elderly population. A detailed description of the EUREYE study design was published previously [19,20]. Briefly, the EUREYE study aimed to enroll 800 to 900 individuals ≥ 65 years of age in each of the 7 participating centers. Participants in the present study were recruited from random sampling of the population aged over 65 registered in the National Office for Statistics Census as residents in Alicante province at the time the sample was requested [20]. The participation rate in the EUREYE study as a whole was 45%, being lower in women and in the older age group. In Spain the response rate for women aged 65–74 was 44.3% and aged ≥ 75 year was 39.2% and for men aged 65 – 74 was 52.4%, and aged ≥ 75 year was 52.7% [20]. Ethical approval for the study was given by the Local Ethical Committee of Sant Joan University Hospital and Miguel Hernández University, Alicante, Spain. Written informed consent was obtained from all subjects.

### Data collection and analysis

On the same day, and prior to the ophthalmic examination, participants were interviewed by trained fieldworkers in the hospital setting, using structure questionnaires for socio-demographic aspects (age, sex, marital status); lifestyle variables (physical activity: sedentary, less active, active, smoking (never, past, current), vitamin supplement use, alcohol consumption, self-referred history of diabetes, and fruit and vegetable intake using a food frequency questionnaire (FFQ). To assess nutritional status Body Mass Index (BMI) was used [21]. BMI was calculated using weight in kilos and height measured to the nearest 0.1 cm with subjects standing without shoes [weight (in kg / height^2)^].The BMI was classified according to the WHO cut-off points for adult population [22] and then re-categorized into obese BMI ≥ 30 kg/m^2^ or non-obese BMI < 30 kg/m^2.^

#### Ophthalmic examination

The ophthalmic examination was performed by experienced ophthalmologists using slit-lamp biomicroscopy. Pupillary dilation was achieved using 0.5% tropicamide and/or 2.5% phenylephrine hydrochloride [20].Cataract was defined as any lens opacity in either eye or evidence of its removal (cataract extraction). A non cataract case was any participant with no evidence of any lens opacities or previous surgery in either eye.

#### Dietary assessment

A semi-quantitative Food Frequency Questionnaire (FFQ) with 135 food items, an expanded and modified version [23] of the Harvard questionnaire [24,25], was used. This FFQ was previously validated in the same geographic region from which the population of the present investigation was drawn. Detailed information on this FFQ was published previously [23]. Nutrient and energy intakes from individual food items in the FFQ were calculated using several food composition tables [26-28]. Nutrient intakes were calculated by multiplying the frequency of use of each food, by the nutrient composition of the portion size and by adding together the nutrient content in all food items to obtain the total nutrient intake for each individual.

#### Plasma antioxidants

Blood samples were sent in dry ice on a monthly basis to a single study’s central accredited laboratory (Queen’s University Belfast) for analysis of blood samples from EUREYE. Carotenoids: lutein, zeaxanthin, β-cryptoxanthin, α-carotene, β-carotene, and lycopene and α-tocopherol were analyzed using a reverse-phase high-performance liquid chromatography method (HPLC) and total ascorbate using an enzyme-based assay in plasma stabilized with metaphosphoric acid [21]. Lutein and zeaxanthin plasma concentrations were combined, as information for these nutrients is given together in main composition food tables. Cholesterol level was also measured using an enzyme assay to adjust concentrations of carotenoids and α-tocopherol [29]. All procedures were verified and validated. All plasma antioxidants were also adjusted according to the season of blood collection (autumn, winter, spring, and summer). The marked seasonality of some products causes wide differences in the supply of fruit and vegetables that act as the main sources of the antioxidants examined, above all beta-cryptoxanthin and lycopene. [30]. In fact, in Spain, fruit and vegetables provide between 3.0 mg (in autumn) and 4.3 mg (in summer) of carotenoids per day [30] and the orange profile presents maximums and minimums, which coincide with those obtained for the serum concentrations of ß-cryptoxanthin [31]. Moreover, in Spain a fairly consistent pattern of carotenoid intake from fresh fruit and vegetables has been observed in the period 1991–2004 [32].

#### Statistical analysis

Individuals, rather than eyes, were the unit of analysis because participants were classified according to the status of the worse eye based on the presence of cataract or cataract surgery against non-cataract. The food, the dietary antioxidants and the plasma antioxidants data were treated separately. Main exposures, fruit and vegetable intake and diet and blood and antioxidants were logarithmically transformed because the skewed distributions and dietary data were adjusted according to the total energy intake model described by Willet et al. [24]. All plasma antioxidants were adjusted according to the season of blood collection (autumn, winter, spring, and summer), and by cholesterol levels except for vitamin C. The standardized values were then categorized into quartiles, with the lowest quartile treated as the reference group. For descriptive purposes, characteristics of participants according to quartiles of fruit and vegetable intake were expressed as percentages or medians (IQR) (Table 1). The Mantel-Haenszel extension chi-square test was used to assess the overall linear trend across increasing quartiles of fruit and vegetable intake and dichotomous variables or ordered categorical variables [33] and the Jonckheere-Terpstra trend test which is a non parametric test, to assess the overall linear trend across increasing quartiles of fruit and vegetables and continuous variables [34] (Table 1). Separate logistic regression analysis was carried out to estimate the association between fruit and vegetable intake, dietary antioxidants, and blood antioxidants and prevalence of cataract. The association between cataract prevalence for each quartile of intake or blood levels was evaluated in terms of OR. For each OR, two-sided P values and 95% CIs were calculated. We used likelihood ratio test (LRT) for exclusion or inclusion of covariates. All covariates with P- values < 0.10 or those reported in the biomedical literature as determinants of cataract were retained in the final models (MODELS III from Tables 2, 3 and 4). Covariates which changed original estimated coefficients (OR) for more than 20% were also included as confounders. The Mantel-extension chi-square test for overall linear was performed to assess the overall trend of odds ratios across increasing quartiles of fruit and vegetables intake, or quartiles of antioxidant vitamins. Statistical analysis was performed using the Stata software package v10 (StataCorp 2007 Stata Statistical Software: Release 10; StataCorp LP, College Station, TX) [35].

**Table 1:** General characteristics of the population according to quartiles of fruit and vegetables intake

		**Quartiles of fruit intake**					**Quartiles of vegetables intake**				
		**[0, 136.2)**	**[136.2, 190.9)**	**[190.9, 271.1)**	**[271.1, 781)**	**p-value**	**[14, 176.2)**	**[176.2, 240.3)**	**[240.3, 322.1)**	**[322.1, 657)**	**p-value**
		n = 150	n = 149	n = 151	n = 149		n = 150	n = 149	n = 151	n = 149	
**Median (IQR)**^**1**^	**Age (years)**	72 (11)	73 (9)	72 (8)	72 (10)	0.938	75 (11)	71 (8)	72 (9)	71 (9)	0.000
**Cataract, (%)**^**2**^	**Yes**	115 (77.7)	112 (75.7)	101 (68.2)	105 (70.5)	0.074	117 (79.1)	107 (72.8)	115 (76.2)	94 (63.9)	0.011
	**No**	33 (22.3)	36 (24.3)	47 (31.8)	44 (29.5)		31 (20.9)	40 (27.2)	36 (23.8)	53 (36.1)	
	**Missings**	2	1	3	0		2	2	0	2	
**Sex (%)**^**2**^	**Men**	83 (55.3)	71 (47.7)	69 (45.7)	51 (34.2)	0.000	77 (51.3)	74 (49.7)	67 (44.4)	56 (37.6)	0.011
	**Women**	67 (44.7)	78 (52.3)	82 (54.3)	98 (65.8)		73 (48.7)	75 (50.3)	84 (55.6)	93 (62.4)	
**Marital status (%)**^**3**^	**Single**	101 (68.2)	97 (65.1)	96 (64.0)	94 (64.4)	0.999	84 (56.4)	98 (65.8)	99 (66.9)	107 (72.8)	0.229
	**Married**	38 (25.7)	42 (28.2)	43 (28.7)	40 (27.4)		52 (34.9)	39 (26.2)	41 (27.7)	31 (21.1)	
	**Separated-divorced**	7 (4.7)	8 (5.4)	9 (6.0)	9 (6.2)		11 (7.4)	8 (5.4)	7 (4.7)	7 (4.8)	
	**Widowed**	2 (1.4)	2 (1.3)	2 (1.3)	3 (2.1)		2 (1.3)	4 (2.7)	1 (0.7)	2 (1.4)	
	**Missings**	2	0	1	3		1	0	3	2	
**Cigarrete Smoking (%)**^**2**^	**Never**	77 (52.0)	82 (55.0)	94 (62.3)	106 (71.1)	0.000	79 (53.0)	89 (59.7)	94 (62.7)	97 (65.1)	0.011
	**Past**	45 (30.4)	41 (27.5)	38 (25.2)	29 (19.5)		41 (27.5)	36 (24.2)	42 (28.0)	34 (22.8)	
	**Current**	26 (17.6)	26 (17.4)	19 (12.6)	14 (9.4)		29 (19.5)	24 (16.1)	14 (9.3)	18 (12.1)	
	**Missings**	2	0	0	0		1	0	1	0	
**Physical activity (%)**^**2**^	**Sedentary**	28 (18.7)	23 (15.4)	27 (17.9)	25 (16.8)	0.065	41 (27.3)	22 (14.8)	27 (17.9)	13 (8.7)	0.000
	**Less active**	104 (69.3)	106 (71.1)	92 (60.9)	92 (61.7)		94 (62.7)	95 (63.8)	105 (9.5)	100 (67.1)	
	**More Active**	18 (12.0)	20 (13.4)	32 (21.2)	32 (21.5)		15 (10.0)	32 (21.5)	19 (12.6)	36 (24.2)	
**Supplement use (%)**^**2**^	**Yes**	22 (14.7)	16 (10.7)	22 (14.7)	25 (16.9)	0.411	26 (17.4)	18 (12.1)	18 (1.9)	23 (15.5)	0.642
	**No**	128 (85.3)	133 (89.3)	128 (85.3)	123 (83.1)		123 (82.6)	131 (87.9)	133 (88.1)	125 (84.5)	
	**Missings**	0	0	1	1		1	0	1	0	
**Diabetes (%)**^**2**^	**Yes**	39 (26.2)	28 (18.8)	22 (14.8)	24 (16.1)	0.017	23 (15.5)	34 (23.0)	27 (17.9)	29 (19.5)	0.645
	**No**	110 (73.8)	121 (81.2)	127 (85.2)	125 (83.9)		125 (84.5)	114 (77.0)	124 (82.1)	120 (80.5)	
	**Missings**	1	0	2	0		2	1	0	0	
**Obesity (%)**^**2**^	**Yes**	51 (34.0)	60 (40.3)	58 (38.4)	50 (33.6)	0.860	50 (33.3)	48 (32.2)	66 (43.7)	55 (36.9)	0.206
	**No**	99 (66,0)	89 (59.7)	93 (61.6)	99 (64.4)		100 (66.7)	101 (67.8)	85 (56.3)	94 (63.1)	
**Median (IQR)**^**1**^	**Energy intake (Kcal)**	1535.8 (562.9)	1526.5 (665.1)	1613.7 (503.7)	1611.5 (500.9)	0.097	1573.5 (587.4)	1600.2 (635.4)	1551.5 (479.6)	1551.2 (572.2)	0.775
	**Alcohol use (grs/d)**	7.1 (13.8)	2.5 (12.5)	0.9 (8.9)	0.5 (5.1)	0.000	1.3 (9.5)	3.5 (13.8)	1.0 (8.9)	1.2 (10.8)	0.409
	**BMI (Kg/m**^**2**^**)**	28.6 (5.4)	29.1 (6.3)	28.8 (6.6)	28.3 (5.1)	0.366	28.1 (5.3)	28.4 (5.2)	29.1 (6.2)	28.9 (5.6)	0.008

Abbreviations: IQR, Interquartile range; BMI, body mass index (calculated as weight in kilograms divided by height in meters squared).

^1^ Jonckheere-Terpstra’s trend test.

^*2*^ Mantel-Haenszel extension chi-square test for overall trend.

^*3*^ Chi-square test.

**Table 2:** **Associations of cataract prevalence with quartiles of fruit and vegetable intake**^**1**^

					***Model I***^***2***^		***Model II***^***3***^	
		***Median intake (grs/d)***	***Number of participants***	***Cases of cataract n(%)***	***OR (95% CI)***	***P-trend***^***4***^	***OR (95% CI)***	***P-trend***^***4***^
**Quartiles of combined fruit and vegetables intake (g/d)**	[15, 356.9)	285.6	148	121 (81.8)	1.00	0.016	1.00	0.008
	[356.9, 453.3)	401.6	148	110 (74.3)	0.66 (0.36 - 1.18)		0.61 (0.33 - 1.12)	
	[453.3, 574.6)	481.4	148	105 (70.9)	0.53 (0.29 - 0.95)		0.45 (0.24 - 0.84)	
	[574.6, 1354)	649.4	149	97 (65.1)	0.44 (0.25 - 0.79)		0.38 (0.20 - 0.70)	
**Quartiles of fruit intake (g/d)**	[0, 136.2)	90.8	148	115 (77.7)	1.00	0.114	1.00	0.024
	[136.2, 190.9)	160.3	148	112 (75.7)	0.81 (0.46 - 1.45)		0.74 (0.41 - 1.34)	
	[190.9, 271.1)	221.1	148	101 (68.2)	0.60 (0.34 - 1.04)		0.57 (0.31 - 1.02)	
	[271.1, 781)	333.7	149	105 (70.5)	0.62 (0.35 - 1.09)		0.56 (0.31 - 1.03)	
**Quartiles of vegetable intake (g/d)**	[14, 176.2)	127.9	148	117 (79.1)	1.00	0.207	1.00	0.135
	[176.2, 240.3)	205.8	147	107 (72.8)	0.95 (0.54 - 1.68)		0.87 (0.48 - 1.59)	
	[240.3, 322.1)	275.0	151	115 (76.2)	1.10 (0.61 - 1.95)		0.95 (0.51 - 1.74)	
	[322.1, 657)	372.1	147	94 (63.9)	0.62 (0.35 - 1.08)		0.54 (0.30 - 0.98)	

^*1*^Odds Ratio (OR) and 95% confidence interval (CI) obtained from logistic regression models.

^*2*^Model I: adjusted for sex, age and energy intake (n=6 missings).

^*3*^Model II: adjusted for factors in model 1and marital status, smoking, alcohol consumption, physical activity, use of supplement, energy intake, obesity (bmi<30 vs ≥30) and history of diabetes (n=20 missings).

^*4*^The Mantel-Haenszel extension chi-square test for overall trend was performed to assess the overall trend of odds ratios across increasing quartiles of fruit and vegetables intake.

**Table 3:** **Associations of cataract prevalence with quartiles of antioxidants intake**^**1**^

					***Model I***^***2***^		***Model II***^***3***^		***Model III***^***4***^	
***Quartiles of intake***		***Median intake***	***Number of***	***Cases of***	***OR (95% CI)***	***P-trend***^***5***^	***OR (95% CI)***	***P-trend***^***5***^	***OR (95% CI)***	***P-trend***^***5***^
		***(mg/d)***	***participants***	***cataract n(%)***						
**Vitamin C (mg/d)**	[12.7, 82.9)	67.0	148	122 (82.4%)	1.00	0.007	1.00	0.006	1.00	0.047
	[82.9, 106.9)	96.0	149	110 (73.8%)	0.64 (0.36 - 1.16)		0.57 (0.31 - 1.05)		0.62 (0.34 - 1.16)	
	[106.9, 143)	122.6	148	102 (68.9%)	0.50 (0.28 - 0.89)		0.46 (0.25 - 0.85)		0.49 (0.27 - 0.92)	
	[143, 408)	173.14	148	99 (66.9%)	0.44 (0.24 - 0.79)		0.40 (0.21 - 0.74)		0.46 (0.24 - 0.88)	
**Vitamin E (μg/d)**	[2.91, 8.09)	7.14	148	126 (85.1%)	1.00	0.016	1.00	0.149	1.00	0.944
	[8.09, 9.27)	8.72	148	99 (66.9%)	0.41 (0.23 - 0.75)		0.40 (0.21 - 0.75)		0.43 (0.23 - 0.82)	
	[9.27, 10.66)	9.84	150	104 (69.3%)	0.42 (0.23 - 0.77)		0.40 (0.21 - 0.75)		0.45 (0.24 - 0.86)	
	[10.66, 22.51)	11.66	147	104 (70.7%)	0.47 (0.25 - 0.86)		0.43 (0.23 - 0.83)		0.49 (0.27 - 0.95)	
**Alfa-carotene (μg/d)**	[21.3, 508.2)	142.96	149	114 (76.5%)	1.00	0.067	1.00	0.036		
	[508.2, 738.9)	401.75	149	114 (76.5%)	0.99 (0.56 - 1.75)		0.90 (0.49 - 1.63)			
	[738.9, 948.3)	809.11	147	103 (70.1%)	0.73 (0.41 - 1.30)		0.72 (0.39 - 1.33)			
	[948.3, 2334)	1153.20	148	102 (64.9%)	0.64 (0.36 - 1.11)		0.55 (0.31 - 1.00)			
**Beta-carotene (μg/d)**	[106, 2845)	2103.76	148	114 (77.0%)	1.00	0.054	1.00	0.020		
	[2845, 4203)	3659.17	150	116 (77.3%)	1.10 (0.62 - 1.95)		1.04 (0.57 - 1.90)			
	[4203, 5425)	4769.32	147	106 (72.1%)	0.91 (0.51 - 1.60)		0.85 (0.47 - 1.55)			
	[5425, 14464)	6557.19	148	97 (65.5%)	0.59 (0.34 - 1.03)		0.53 (0.30 - 0.95)			
**Cryptoxanthin (μg/d)**	[2.97, 146.93)	108.57	149	118 (79.2%)	1.00	0.312	1.00	0.103		
	[146.93, 205.12)	173.61	147	105 (71.4%)	0.65 (0.37 - 1.15)		0.67 (0.37 - 1.20)			
	[205.12, 302.63)	251.45	148	104 (70.3%)	0.55 (0.31 - 0.98)		0.57 (0.32 - 1.04)			
	[302.63, 1175)	404.50	148	106 (71.1%)	0.69 (0.39 - 1.23)		0.69 (0.38 - 1.25)			
**Lycopene (μg/d)**	[0, 870)	582.72	149	114 (76.5%)	1.00	0.816	1.00	0.616		
	[870, 1279)	1051.05	147	108 (73.5%)	0.88 (0.50 - 1.55)		0.87 (0.49 - 1.57)			
	[1279, 1981)	1525.04	148	102 (68.9%)	0.70 (0.40 - 1.22)		0.66 (0.37 - 1.18)			
	[1981, 19518)	2706.69	149	109 (73.2%)	0.88 (0.50 - 1.54)		0.93 (0.51 - 1.68)			
**Lutein-zeaxanthin (mg/d)**	[114, 2239)	1584.09	150	116 (77.3%)	1.00	0.517	1.00	0.820		
	[2239, 3162)	2647.72	147	106 (72.1%)	0.93 (0.53 - 1.64)		0.82 (0.45 - 1.47)			
	[3162, 4380)	3680.51	148	106 (71.6%)	0.90 (0.52 - 1.57)		0.78 (0.44 - 1.39)			
	[4380, 18633)	5397.06	148	105 (70.9%)	0.86 (0.49 - 1.49)		0.76 (0.43 - 1.36)			

^*1*^Odds Ratio (OR) and 95% confidence interval (CI) obtained from logistic regression models.

^*2*^Model I: Adjusted for sex, age and energy intake (n=6 missings).

^*3*^Model II: Adjusted for factors in Model I and marital status, smoking, alcohol consumption, physical activity, use of supplement, energy intake, obesity (BMI<30 vs ≥30) and history of diabetes (n=20 missings).

^4^Model III: Adjusted for factors in Model II plus the antioxidants whose p-value ≤ 0,10, for model of vitamin C adjusted for vitamin E and viceversa for model of Vitamin E, adjusted for vitamin C, (n=20 missings).

^*5*^The Mantel-Haenszel extension chi-square test for overall trend was performed to assess the overall trend of odds ratios across increasing quartiles of fruit and vegetables intake.

**Table 4:** **Associations of cataract prevalence with quartiles of plasma antioxidants**^**1**^

					***Model I***^***2***^		***Model II***^***3***^		***Model III***^***4***^	
**Quartile of blood level**		***Median intake***	***Number of***	***Cases of***	***OR (95% CI)***	***P-trend***^***5***^	***OR (95% CI)***	***P-trend***^***5***^	***OR (95% CI)***	***P-trend***^***5***^
		***(μmol/L)***	***participants***	***cataract***						
**Vitamin C (*μmol/L)***	[2.00, 33.5)	19.00	137	108 (78.8%)	1.00	0.077	1.00	0.169	1.00	0.553
	[33.5, 45.6)	40.70	139	109 (78.4%)	1.11 (0.61 - 2.02)		1.23 (0.65 - 2.31)		1.20 (0.63 - 2.28)	
	[45.6, 58.6)	51.90	138	81 (58.7%)	0.41 (0.23 - 0.73)		0.41 (0.23 - 0.74)		0.39 (0.21 - 0.72)	
	[58.6, 127.9)	67.55	136	102 (75.0%)	0.80 (0.44 - 1.48)		0.97 (0.51 - 1.84)		1.02 (0.53 - 1.99)	
**Alfa-tocopherol (*μmol/L)***	[13.78, 28.24)	26.08	137	105 (76.6%)	1.00	0.026	1.00	0.624	1.00	0.927
	[28.24, 31.22)	29.84	139	110 (79.1%)	1.06 (0.58 - 1.93)		1.03 (0.57 - 1.99)		0.92 (0.48 - 1.75)	
	[31.22, 34.99)	32.03	137	88 (64.2%)	0.41 (0.23 - 0.73)		0.42 (0.23 - 0.76)		0.37 (0.20 - 0.69)	
	[34.99, 53.19)	37.60	138	98 (71.0%)	0.68 (0.38 - 1.21)		0.65 (0.36 - 1.19)		0.51 (0.27 - 0.96)	
**Alfa-carotene (*μmol/L)***	[0.00, 0.03)	0.02	137	106 (77.4%)	1.00	0.604	1.00	0.223		
	[0.03, 0.05)	0.04	139	96 (69.1%)	0.62 (0.35 - 1.10)		0.66 (0.36 - 1.20)			
	[0.05, 0.09)	0.06	137	103 (75.2%)	1.03 (0.57 - 1.85)		0.98 (0.53 - 1.81)			
	[0.09, 1.38)	0.15	138	96 (69.6%)	0.69 (0.39 - 1.25)		0.60 (0.32 - 1.13)			
**Beta-carotene (*μmol/L)***	[0.00, 0.10)	0.06	138	104 (75.4%)	1.00	0.685	1.00	0.118		
	[0.10, 0.16)	0.12	138	93 (67.4%)	0.64 (0.37 - 1.13)		0.71 (0.39 - 1.28)			
	[0.16, 0.29)	0.22	137	102 (74.5%)	1.01 (0.56 - 1.82)		1.14 (0.62 - 2.11)			
	[0.29, 3.19)	0.47	138	102 (73.9%)	0.97 (0.54 - 1.76)		1.02 (0.55 - 1.90)			
**Beta-cryptoxanthin (*μmol/L)***	[0.00, 0.04)	0.02	137	105 (76.6%)	1.00	0.741	1.00	0.347		
	[0.04, 0.07)	0.05	138	93 (67.4%)	0.58 (0.33 - 1.03)		0.61 (0.33 - 1.11)			
	[0.07, 0.15)	0.10	139	102 (73.4%)	0.81 (0.45 - 1.45)		0.90 (0.48 - 1.67)			
	[0.15, 1.11)	0.26	137	101 (73.7%)	0.77 (0.42 - 1.41)		0.77 (0.40 - 1.46)			
**Zeaxanthin (*μmol/L)***	[0.00, 0.02)	0.01	138	104 (75.4%)	1.00	0.493	1.00	0.542		
	[0.02, 0.03)	0.02	138	102 (73.9%)	0.96 (0.54 - 1.70)		1.15 (0.62 - 2.11)			
	[0.03, 0.05)	0.03	138	94 (68.1%)	0.68 (0.38 - 1.20)		0.77 (0.43 - 1.40)			
	[0.05, 0.21)	0.07	137	101 (73.7%)	0.89 (0.48 - 1.63)		0.93 (0.49 - 1.76)			
**Lycopene (*μmol/L)***	[0.00, 0.20)	0.11	137	111 (81.0%)	1.00	0.580	1.00	0.074		
	[0.20, 0.37)	0.28	139	90 (64.7%)	0.41 (0.23 - 0.74)		0.41 (0.22 - 0.76)			
	[0.37, 0.68)	0.49	137	97 (70.8%)	0.58 (0.32 - 1.05)		0.69 (0.37 - 1.30)			
	[0.68, 6.79)	1. 09	138	103 (74.6%)	0.68 (0.37 - 1.28)		0.72 (0.38 - 1.38)			
**Lutein-zeaxanthin (*μmol/L)***	[0.00, 0.05)	0.04	138	101 (73.2%)	1.00	0.548	1.00	0.138		
	[0.05, 0.08)	0.06	137	105 (76.6%)	1.37 (0.77 - 2.44)		1.41 (0.78 - 2.58)			
	[0.08, 0.21)	0.12	138	95 (68.8%)	0.81 (0.46 - 1.43)		0.86 (0.47 - 1.54)			
	[0.21, 1.17)	0.31	138	100 (72.5%)	0.92 (0.51 - 1.67)		0.91 (0.48 - 1.71)			

^*1*^Odds Ratio (OR) and 95% confidence interval (CI) obtained from logistic regression models.

^*2*^Model I: Adjusted for sex, age and season (n=48 missings)

^*3*^Model II: Adjusted for factors in model I and marital status, smoking, alcohol consumption, physical activity, use of supplement, obesity (bmi<30 vs ≥30) and history of diabetes (n=60 missings)

^4^Model III: Adjusted for factors in Model II and the plasma antioxidants whose p-value≤0,10: Vitamin C and Alpha-tocopherol (n=60 missings).

^*5*^The Mantel-Haenszel extension chi-square test for overall trend was performed to assess the overall trend of odds ratios across increasing quartiles of fruit and vegetables intake.

## Results

Of 599 elderly people recruited, 593 individuals were eventually included in the analyses, 319 women (54%) and 274 men (46%), since 6 participants were excluded due to missing data. Of these, 433 (73%) had cataract, being 54% women (n = 234), and 46% men (n = 199). There were no significant differences for median intakes of fruit between cataract cases compared to non cataract (186.g/day vs. 200.g/day p = 0.069), although for median intakes of vegetables a significant difference was observed (234 g/day vs. 259 g/day p = 0.004, respectively). General characteristics of the population according to quartiles of fruit and vegetable intake are shown in Table 1. Those with higher vegetable intake were women, slightly younger, single, non-smokers, who were physically active. Additionally, those with a higher fruit intake tended to be women, non-smokers, with lower alcohol consumption and were mainly non-diabetic.

The odds ratios (OR) and 95% confidence intervals (CI) for cataract obtained from logistic regression models first across quartiles of combined fruit and vegetables and then separately from quartiles of fruit followed by quartiles of vegetables are shown in Table 2. There were significant differences between median intakes of combined fruit and vegetables between participants with cataract versus participants without cataract (444 g/day vs. 488 g/day p = 0.001, respectively). Increasing quartiles of combined fruit and vegetable intake adjusted for age sex and energy (Model I) were significantly associated with decreasing odds of prevalent cataract (*P* for trend = 0.016, Table 2). After further adjustments for marital status, smoking, alcohol consumption, physical activity, supplement use, obesity and history of diabetes, a similar association was observed between increasing quartiles of intake (*P for trend* = 0.008). No association was found in separate analyses of intakes of fruit or vegetables and prevalence of cataract.

Table 3 shows associations of each dietary antioxidant and prevalence of cataract. First, separated models with each antioxidant adjusted for age-sex and energy are presented in Model I, followed by the same model with further adjustments (Models II, & III). Thus, increasing quartiles of daily dietary intakes from 107 mg/d of vitamin C were associated with decreasing prevalence of cataract (*P* for trend = 0.047) (Model III). Dietary vitamin E, from daily intakes of 8 mg/d (second quartile)*,* was associated with decreased prevalence of cataract, although no linear trend was observed across quartiles (*P* for trend = 0.944) (Model III). No other dietary antioxidants were associated with cataract.

Table 4 shows the equivalent results for the blood antioxidants. The OR relating the prevalence of cataract in the third and fourth quartile of plasma α-tocopherol to that of the lowest quartile was statistically significant OR = 0.37 95% CI (0.20 – 0.69) and OR = 0.51 95% CI (0.27 -0.96) respectively, but the inverse trend across quartiles was non significant (*P* for trend = 0.927). No association was seen for any other plasma antioxidant and prevalence of cataract or previous cataract surgery.

## Discussion

Results from this study showed that increasing quartiles of combined fruit and vegetable intake and increasing quartiles of dietary vitamin C were associated with a significant inverse trend of cataract prevalence or previous cataract surgery. In addition, levels from second quartile of dietary vitamin E, and third quartile of plasma levels of α-tocopherol were associated with reduced odds of prevalent cataract, although no linear trend was observed. No other association was found between any other antioxidant vitamins and prevalence of cataract in this study.

Previous studies which have looked at fruit and vegetable intake have found only a non significant or a modest association with cataract risk [11,16,17]. For instance, a cross-sectional analysis of 479 women aged 52–73, participants from the Harvard Nurses’ Health Cohort Study, showed a non-significant reduced odds of prevalent nuclear opacities (n = 163) for those in the highest quartile of fruit intake (median: 3.9 servings) versus low intake (median: 1.3 servings) (OR = 0.58; 95% CI = 0.32-1.05), *P* for trend = 0.31 [16]. Similarly, cross-sectional data from the Beaver Dam Eye Study with 1919 participants aged 43–84, found that a high intake of fruit and vegetables (fifth quintile; median: 4.9 servings) was associated with a non-significant reduced odds of prevalent nuclear sclerosis in men in the low quintile (median: 2.3 servings) (OR = 0.68; 95% CI = 0.43-1.09), *P* for trend = 0.07, but not in women [11]. Prospective data in that cohort, based on 5 years of follow-up, indicated a non-significant reduction in risk of cataract for those with a high intake of vegetables at baseline (and a possible elevated risk of cataract for those with high fruit intake) [11]. The Women’s Health Study (WHS) is the largest prospective study on cataract in relation to total fruit and vegetable intake [36]. During an average of 10 years of follow-up, 2067 cases of incident cataract and 1315 cases of cataract extraction were confirmed. Compared with women in the lowest quintile of fruit and vegetable intake, those in quintiles 2–5 (≥ 3,4 servings/day) had a moderate (10%–15%) reduced risk of cataract *P* for trend = 0.05.

In the present study, a strong inverse association was found between increasing quartiles of combined fruit and vegetables intake and the prevalence of cataract. These intakes were found to be notably higher when compared with other studies. In fact, fifty percent of this population fulfilled the WHO recommendations of 5 or more servings of fruit and /or vegetables a day (> 400 g/day), median 440 g/d, (IQR 226). The Alicante diet is a Mediterranean type of diet, which is rich in fruit and vegetables, specifically in citrus fruit, and hence, provides high concentrations of antioxidant vitamins [37].

Among the antioxidants examined, dietary vitamin C showed a more consistent effect on cataract prevalence. Our results suggest that daily dietary vitamin C intakes above 107 mg/d were inversely associated with reduced odds of cataract prevalence (*P* for trend among the fourth quartiles = 0.047). Compared to the lowest quartile intakes between 13 mg/d to 83 mg/d, a significant 38% lower odds for cataract prevalence was found for intakes between 83 mg/d and 107 mg/d, a 51% lower odds for intakes between 107 and 143 mg/d, and 54% lower odds for intakes between 143 and 408 mg/d. These data are consistent with previous work which found that human eye tissues saturate at intakes of vitamin C between 200 and 300 mg/d [38]. Caution is required in comparing dietary intake levels across studies because of the variation in dietary assessment methods and food composition tables. In relation to dietary vitamin C, results of the present work are similar to those found in the Nutrition and Vision Project (NVP) [39], which was based on a subset of women from the Nurses’ Health Study. The NVP observed a significant 48% decreased odds of nuclear opacities for intakes between 140 mg/d and 180 mg/d, a 53% reduced odds for intakes between 180 mg/d to 240 mg/d, and 66% reduced odds for intakes between 240 mg/d and 360 mg/d compared to the lowest quintile intakes < 140 mg /d. In the original Nurses Health cohort study duration of vitamin C supplement use was strongly associated with a reduced rate of cataract extraction [10], but in a later expanded analysis with a longer follow-up no relation was seen between duration of supplement use of vitamin C and cataract extraction, although a lower risk with long term vitamin C supplement use was suggested [40]. Inverse associations have also been reported in other studies [41-43] but not in all [15,44]. In the present work, the results for dietary vitamin C were not supported by a significant trend in plasma levels of ascorbic acid (mean = 45.04 μmol/L; SD ± 20.02). In fact, plasma ascorbic acid levels from 46 μmol/L to 59 μmol/L (third quartile) showed a significant 60% reduced odds of cataract prevalence compared to those in the lowest quartile < 33 μmol/L, but higher levels above 59 μmol/L showed no effect. Results from other studies are not consistent. In the NHANES II study [45], a 1 mg/dL increase in serum vitamin C (equivalent to a 58 μmol/L increase) was associated with a 26% reduced risk in self-reported cataract extraction. The average vitamin C levels were 62 μmol/L (SD = 28). In a case–control study carried out in southern Spain, levels of plasma ascorbic acid above 49 μmol/L were associated with a 64% reduced odds of cataract compared to levels ≤ 49 nmol/L (*P* for trend among the five quintiles < 0.0001 among the five groups) [37]. In another southern European study, a significant inverse association was found for those in the highest quintile ( > 70 μmol/L) compared to those in the lowest quintile < 30 μmol/L [46]. In the NVP [39], the OR relating the prevalence of nuclear opacities in the fourth ascorbic acid quintile, between 75 μmol/L and 86 μmol/L, compared to that in the lowest < 50 μmol/L, was statistically significant, but the OR in the highest quintile was only marginally significant. No association between plasma vitamin C and cataract was found in the POLA study in southern France (mean vitamin C ~ 35 μmol/L [47] or in the United Kingdom, for levels > 57 μmol/L compared to < 24 μmol/L [48]. In the Baltimore longitudinal study, no association was found between levels of ascorbic acid from 82 μmol/L compared to levels < 60 μmol/L [49].

In the present study, both daily dietary intakes from 8 mg /d of vitamin E (second quartile) and blood levels from 31 μmol/l (third quartile) were inversely associated with reduced odds of cataract prevalence although no linear trend was observed. Until now, epidemiological studies examining vitamin E and cataract have produced conflicting results. In the NVP, inverse associations between nuclear opacities and vitamin E intake, supplement use and duration of use of vitamin E, and supplement use of multivitamins, were observed, although authors were not totally certain that these associations were independent of vitamin C relationship [39]. In the longitudinal study of cataract [44], multivitamin and vitamin E supplement use was inversely associated with risk of nuclear opacification. However, other observational studies have not found an association between cataract and vitamin E intake [37,50]. Although the nutritional status of vitamin E has been estimated by measuring plasma concentrations of α-tocopherol, which reflects intake in well-nourished subjects [25], results from epidemiological studies about the relation between plasma α-tocopherol levels and cataract are not consistent. Some studies have found an inverse association either with high α-tocopherol levels [44,51], or with total serum tocopherols (the sum of alpha- and gamma-tocopherol), [14]. However, none of these studies measured vitamin C levels in blood and, therefore, could not evaluate the independent association with α-tocopherol. Among those which measured vitamin C and found a protective effect, the Beaver Dam cohort study [14] found an inverse association between plasma vitamin E and incidence of nuclear opacities and in the NVP study [39], plasma α-tocopherol levels were associated with reduced odds of nuclear opacities. The Baltimore study, with similar plasma α-tocopherol levels to those found in the present study, showed a 45% decreased odds for nuclear cataract for medium quartile levels between 18.57 μmol/l and 29.72 μmol/l, and a 48% decreased odds for the highest quartile levels >29.720 μmol/l compared to the lowest quartile [49]. However, other studies have not found an association with α-tocopherol levels [37,52] and one reported an increased risk of cortical and PSC with high levels of α-tocopherol [46].

No other antioxidant examined in this study showed an association with cataract prevalence. Lutein and zeaxanthin are the main carotenoids found in the lens, but very little β–carotene is found, and no lycopene has been found [53]. Results from the NVP [39] suggest that women with luthein/zeoxanthin intake above 2.4 mg/d may have a lower risk of nuclear opacities, although this association was not clearly independent of the relation of vitamin C. Results from the Nurses Health Study [40] and the Health Professional Follow-up study [13] showed that women and men with lutein/zeaxanthin intakes of 4 to 6 mg/d had reduced rates of cataract extraction. The Beaver Dam Eye study found a strong decrease of 30 to 40% in reduction of risk of nuclear opacities [14]. Three studies examined plasma lutein and zeaxanthin [43,48,54], but only one found a significant inverse association [54]. Of the studies that investigated β–carotene, [37,46,48,49] only one found an inverse association [48]. Two studies found an inverse association between high dietary and blood levels of lycopene and cataract [10,11,37].

When interpreting the results of the present work, several possible limitations should be considered. First, the low response rate of 50% [20], could have affected the estimates, but it is unlikely that the reason for participating in this study was related to the main risk factors under examination, the intake of fruit and vegetables and antioxidant vitamins.

Several reasons lead to this supposition. At the time of inclusion, medical practice in Spain did not include dietary advice to patients with cataract, and potential participants did not know the objectives under investigation. On the other hand, the fact that mean blood values for α-tocopherol and ascorbic acid, a measure which is less susceptible to error, were within the range found in other Spanish studies suggests that there was less likelihood of selection bias. In this study mean value for α-tocopherol for women was 33 μmol/L, similar to two other Spanish studies with mean value of 34 μmol/L and 33 μmol/L [31,37]. For men mean value was 30 μmol/L, which was slightly lower compared to the other two Spanish studies with mean value of 32 μmol/L and 32 μmol/l. [31,37]. For ascorbic acid mean serum concentrations was 0.79 mg/dl, (median 0.80 mg/dl), which was slightly lower when compared with another southern Spanish population, with a mean of 1.00 mg/dl (median = 1.01 mg/dl) [37]. However, comparison between participants and non-participants in the present study was not possible because the mode of contact in the EUREYE study was restricted by ethical committees. People who refused to participate could not be re-contacted, and therefore reasons for refusal could not be collected [20]. The limitations in this study also include the fact that a single measurement of intake increases the possibility of measurement error of intakes, diluting the associations. In the NVP study [39], similar associations between nuclear opacities and vitamins were observed, when the effect of characterizing the intake with 5 measurements of intake over 13 to 15 years was compared to the effect of a single value obtained from a single measure from the end of this interval. Therefore, if misclassification of intakes from the FFQ occurred, these would be expected to lead to a dilution of the magnitude of the associations. Of more concern is recall bias, where errors in reporting food intakes occur because of biased reporting due to knowledge of being a case. However, this seems unlikely. At the time of the study, there was little awareness in the population of possible relation between diet and cataract, and questionnaires were administered prior to the ophthalmic examination. We could examine this partially. Of the 593 individuals included in the analyses, 17.5% (n=104) had undergone cataract surgery at inclusion in the study. Of these, 26.9% (n= 28) had received cataract surgery on one eye and 73.0% (n=76) on both eyes*.* The surgery cases would have been well aware of their having had a cataract extraction, and this may have influenced their subsequent diet or reporting of their diet. However, when comparisons were made between mean intakes of fruit and vegetables in prevalent surgery cases compared to prevalent cataract cases, no statistical differences were found: mean intake for surgery cases was 453 mg/d (± 76) compared to mean value for cataract prevalent cases which was 447 (± 183), p = 0.765. In this study, only two antioxidant vitamins, vitamins C and E, were associated to decreased cataract prevalence. It is likely, however, that due to the strong correlation between nutrients, the ability of this study to identify the individual role of the different antioxidants may have been limited. In fact, strong correlations between most of the nutrients were found with Spearman correlation coefficients approaching or exceeding 0.50, which indicates the need to consider the relations between nutrients. Final models III in Tables 3 &4, were performed adjusted for all antioxidants, and repeated only considering those which remained significantly associated to cataract prevalence, i.e. vitamins C and E, but the estimates in these models remained the same. Although the possibility of residual confounding could not be ruled out completely, the associations between vitamins C and E intake were examined under the most conservative adjustments taking into account a large number of likely or known determinants of cataract. Results from dietary vitamins C and E were not completely supported by blood results of these antioxidants. The Spearman correlation coefficients between intake of Vitamins C and E and their blood levels were weak, r = 0.25 and r = 0.05 respectively. This lack of correlation might have been due to high intra person variability in blood ascorbic acid and α-tocopherol levels. Classified participants about their antioxidant blood levels accurately based on a single blood analysis might have led to a misclassification error. A protective role of vitamins C and E on cataract formation is biologically plausible. Ascorbic acid and α-tocopherol are powerful reducing agents which protect the eye from oxidative stress. Vitamin C, is the most important antioxidant vitamin found in the lens at concentrations of 30-to 50-fold that of plasma [52]. α-tocopherol, is found in human lens at the same levels as in plasma. Both vitamins C and E function at their own eye sites individually and also synergistically. Vitamin C is known to renew the reduced state of tocopherol and both vitamins maintain the antioxidant activity of glutathione [55]. Glutathione is the essential and primary lenticular antioxidant. The loss is apparently due to the oxidation of glutathione (GSH) to GSSG since its levels rise significantly once the cataract develops. The loss of GSH occurs in nearly all experimental cataract [56].

Finally, findings for the different antioxidants examined in this study are not consistent with other epidemiological studies. Differences might be explained by variations in the population characteristics, study design, and the methods used to assess intake of antioxidant levels. Additionally, genetic variants that influence the blood levels of antioxidants have been identified recently. An association between the heterozygote allele of the SCARBI gene, which is involved in the uptake of vitamin E and lutein, and macular degeneration was observed in two populations in a case control study in France and the USA [57]. So far there is no evidence of an association between variants influencing antioxidant blood levels and cataract risk, but this should be also examined in future studies.

## Conclusions

The results of this study support WHO recommendations regarding the beneficial effect of daily intakes of 5 or more portions of fruit and vegetables on the risk of non-communicable diseases, including cataract. Even in this population, with high intakes of fruit and vegetables, those with levels of fruit and vegetables < 453 g /d (50%) or levels of vitamin C intake < 107 mg /d (50%) or vitamin E intake < 8 mg / d (25%) had higher prevalence of cataract. Results of this study also support the important role of the antioxidant vitamins on cataract formation. Further understanding of both, the individual role of the antioxidants, and their synergistic effects is needed. Studies in diverse populations with longer follow-ups, to examine other factors which might influence the relationship between diet and cataract, including genetic factors, should be carried out. These studies should also assess the relation between oxidative and inflammatory processes before and after the intervention or follow-up, and should measure the efficacy of the antioxidant dose in the reduction of the level of these damages.

## Competing interests

The author declares that she has no competing interests.

## Pre-publication history

The pre-publication history for this paper can be accessed here:

http://www.biomedcentral.com/1471-2415/13/52/prepub
